# Country-Specific Data and Priorities for Pertussis in Latin America: Recent Findings From the Global Pertussis Initiative

**DOI:** 10.1093/ofid/ofaf154

**Published:** 2025-05-13

**Authors:** Rolando Ulloa-Gutierrez, Daniela Hozbor, María L Avila-Aguero, Gabriela Echániz-Aviles, Angela Gentile, Juan Pablo Torres Torretti, Ulrich Heininger, Rudzani Muloiwa, Carl Heinz Wirsing von König, Kevin Forsyth, Tina Q Tan

**Affiliations:** Servicio de Aislamiento, Hospital Nacional de Niños Dr Carlos Sáenz Herrera, Caja Costarricense de Seguro Social, San José, Costa Rica; Cátedra de Padriatria, Universidad de Ciencias Médicas, San José, Costa Rica; Laboratorio VacSal, Instituto de Biotecnología y Biología Molecular, Facultad de Ciencias Exactas, Universidad Nacional de La Plata, National Scientific and Technical Research Council (CONICET) La Plata, Argentina; Servicio de Infectología, Hospital Nacional de Niños, San José, Costa Rica; Researcher Affiliated with the Center for Modeling and Analysis of Infectious Diseases, Yale University, New Haven, Connecticut, USA; Center for Research on Infectious Diseases, Instituto Nacional de Salud Pública, Cuernavaca, Mexico; Hospital de Niños Ricardo Gutiérrez, University of Buenos Aires, Buenos Aires, Argentina; Departamento de Pediatría, Hospital Dr Luis Calvo Mackenna, Facultad de Medicina, Universidad de Chile, Santiago, Chile; Pediatric Infectious Diseases, University of Basel Children's Hospital, Basel, Switzerland; Department of Paediatrics and Child Health, Faculty of Health Sciences, University of Cape Town, Cape Town, South Africa; Independent Researcher, Krefeld, Germany; College of Medicine and Public Health, Flinders University, Adelaide, South Australia, Australia; Feinberg School of Medicine, Northwestern University, Chicago, Illinois, USA

**Keywords:** Global Pertussis Initiative, immunization, Latin America, pertussis, surveillance

## Abstract

In 2023, the Global Pertussis Initiative met to assess the burden of and vaccination policies against pertussis in 10 Latin American countries. Although pertussis is a notifiable disease in the represented countries, poor disease awareness, underrecognition in older individuals, and limited laboratory capacity and supplies challenge the collection of robust epidemiological data. Infants in all 10 countries receive a 3-dose primary series followed by ≥2 boosters. Except for Paraguay and Venezuela, governments of the represented countries advise or mandate vaccination in pregnancy; however, coverage rates remain suboptimal. Healthcare providers and the public should be educated on how mothers and other contacts can serve as asymptomatic carriers of *Bordetella pertussis* (transmitting disease to vulnerable infants) and of the potentially unique presentation of pertussis in adolescents and adults. The burden of pertussis in Latin America can be reduced by improving vaccination coverage of the primary series, increasing vaccination in pregnancy, and instituting universal vaccination of adults.

## Teaser

This article provides a comprehensive analysis of pertussis epidemiology, surveillance strategies, and vaccination policies in Latin America. It highlights critical vaccine coverage gaps—particularly in all recommended doses and during pregnancy—emphasizing the urgent need for targeted strategies to enhance disease control.

Cases of pertussis (whooping cough) have been increasing in many countries in recent decades [1–6]. Pertussis is often perceived as a childhood disease—indeed, infants are at the highest risk of severe outcomes—but people of any age can be affected [1, 3, 7–12]. In recognition of this, an expert scientific forum known as the Global Pertussis Initiative (GPI) was launched in 2001 to assess global trends in and raise awareness of pertussis [13].

Vaccination remains the most effective strategy to control pertussis [14], and the GPI endorses several immunization strategies. These include strengthening coverage rates for the primary vaccination series; introducing universal booster doses for preschoolers, adolescents, and adults; promoting vaccination in pregnancy (ViP); and, where ViP is not practiced, selectively immunizing those who come into close contact with unimmunized infants, such as parents, other household contacts, and childcare workers (ie, cocooning) [13, 15–17]. However, the need and ability to augment or expand vaccination policies must be considered in light of local disease epidemiology; political will, including competing health priorities; and financial resources.

Despite long-standing vaccination programs, many Latin American countries began to experience increases in pertussis cases in the 1990s, with several outbreaks reported since the 2000s [18–21]. To address the challenges of pertussis in Latin America, the GPI convened meetings focused on this region in 2008 [22] and 2017 [18]. In 2023, a third meeting was held in Buenos Aires, Argentina, with GPI steering committee members and 16 experts from 10 Latin American countries: Argentina, Brazil, Chile, Colombia, Costa Rica, Mexico, Paraguay, Peru, Uruguay, and Venezuela. The objectives of this meeting were to (1) share information on the epidemiology, surveillance, diagnosis, and awareness of pertussis in Latin America since the 2017 GPI meeting; (2) describe current (and potentially propose new) pertussis vaccination recommendations, with a focus on pregnant people, adolescents, and adults; and (3) discuss the impact of the coronavirus disease 2019 (COVID-19) pandemic on vaccination recommendations and vaccine coverage rates (VCRs).

## SURVEILLANCE AND DIAGNOSIS

Pertussis is a notifiable disease in all 10 Latin American countries represented at the 2023 GPI meeting. Clinical case definitions of pertussis vary across countries, with some (eg, Argentina, Brazil, Chile, Colombia, Mexico, Peru) employing age-based clinical case definitions (Table 1) [23–30]. All countries use polymerase chain reaction (PCR) to confirm suspected clinical cases of pertussis. Culture may also be used for laboratory confirmation (Table 1).

**Table 1.** Case Definitions of Pertussis Used in Select Latin American Countries

	Argentina [22]	Brazil [23]	Chile [24]	Colombia [25]	Costa Rica [26]	Mexico [27]	Paraguay [28]	Peru [29]	Uruguay
Clinical	<6 mo: Acute respiratory infection accompanied by paroxysms, inspiratory stridor, posttussive vomiting, apnea, and/or cyanosis	<6 mo: (irrespective of vaccination status): Cough lasting ≥10 d accompanied by paroxysms, inspiratory whoop, posttussive vomiting, choking, apnea, and/or cyanosis	<6 mo: Cough and coryza, afebrile or with low-grade fever, accompanied by inspiratory stridor, posttussive vomiting, apnea, cyanosis, and/or paroxysmal cough	<3 mo: Acute respiratory infection accompanied by paroxysmal cough, inspiratory whoop, posttussive vomiting, apnea, and/or cyanosis	…	<6 mo: Acute respiratory infection (irrespective of duration) accompanied by apnea and/or cyanosis	…	<3 mo: Nonspecific clinical signs of an upper respiratory tract infection associated with persistent cough, apnea, and/or cyanosis	Cough lasting ≥14 d
	6 mo to 11 y: Cough lasting ≥14 d accompanied by paroxysms, inspiratory stridor, and/or posttussive vomiting without other apparent cause	≥6 mo (irrespective of vaccination status): Cough lasting ≥14 d accompanied by paroxysms, inspiratory whoop, and/or posttussive vomiting	6 mo to 9 y: Paroxysms of cough lasting ≥7 d, afebrile or with low-grade fever accompanied by inspiratory stridor, apnea, and/or posttussive vomiting in the absence of another apparent cause	3 mo to 12 y: Severe paroxysmal cough of any duration accompanied by stridor or, in the absence of a more likely diagnosis, an acute respiratory infection with a cough lasting >7 d that is accompanied by paroxysms, stridor, and/or posttussive vomiting	<1 y: Apnea of any duration accompanied by paroxysms, inspiratory noise or cry, and/or vomiting after coughing or vomiting without other apparent cause	Cough lasting ≥14 d accompanied by paroxysms, inspiratory stridor, and/or posttussive vomiting or hemorrhage (conjunctival, petechial, or epistaxis)	<1 y: Cough of any duration, without any apparent cause, accompanied by paroxysmal cough, inspiratory stridor, posttussive vomiting or vomiting without any apparent cause, and/or apnea; alternatively, clinical suspicion of pertussis	≥3 mo: Persistent cough lasting ≥2 wk accompanied by paroxysms, posttussive vomiting, and/or rales on inspiration	Paroxysmal cough of any duration accompanied by posttussive vomiting, apnea, cyanosis, stridor, and/or seizures
	>11 y: Persistent cough lasting ≥14 d in the absence of other symptoms	…	>9 y: Nonproductive, paroxysmal cough lasting ≥14 d plus inspiratory stridor, apnea, sweating between coughing episodes, posttussive vomiting, and/or worsening of cough at night	>12 y: Respiratory infection accompanied by a cough lasting >14 d, irrespective of the presence of paroxysms, expectoration, or posttussive vomiting	Any age: Paroxysmal cough lasting ≥2 wk	…	>1 y: Cough lasting ≥2 wk, without any apparent cause, accompanied by paroxysmal cough, inspiratory stridor, and/or posttussive vomiting or vomiting without any apparent cause; alternatively, clinical suspicion of pertussis	Adolescents and adults: Persistent cough lasting ≥2 wk without “whining” as symptoms that mimic bronchitis or asthma, or as the classical symptoms of whooping cough	…
Lab-confirmed	PCR, culture, serology	rt-PCR, culture	PCR, culture, serology	PCR, culture	rt--PCR	rt--PCR, culture, serology	PCR, culture	PCR, culture	PCR, culture, serology
Epidemiological	Link to a lab-confirmed case 3–21 d before symptom onset	Close contact with a lab-confirmed case	Contact with a lab-confirmed case	Contact with a lab-confirmed case	…	Contact with respiratory secretions from a probable or confirmed case	Link to a lab-confirmed case in the 3 wk before symptom onset	Link to a lab-confirmed case	Contact with a suspected or confirmed case that lacks other apparent cause

Due to the lack of information, Venezuela was not included in the table.

Abbreviations: lab, laboratory; PCR, polymerase chain reaction; rt--PCR, real-time polymerase chain reaction.

### Argentina

Pertussis has been a notifiable disease in Argentina since 1960, with cases reported to the National Health Surveillance System Clinical Surveillance Module since 2009. There are 2 reference laboratories in Argentina: 1 in Buenos Aires (National Institute of Infectious Diseases, which is part of the National Administration of Laboratories and Institutes of Health “Dr Carlos G. Malbrán”) and 1 in La Plata (National Pertussis Reference Laboratory in Argentina, La Plata Headquarters). At these laboratories, suspected clinical cases of pertussis can be confirmed by culture, real-time PCR (rt-PCR), and serology.

### Brazil

A notifiable disease since 1973, pertussis cases in Brazil are reported to the National Notifiable Diseases Information System. Through a sentinel network composed of 34 general and pediatric hospitals, pertussis is actively monitored in São Paulo. Clinical sample testing is performed at the national reference laboratory (Adolofo Lutz Institute in São Paulo) or at 1 of 12 state reference laboratories.

### Chile

Suspected clinical cases of pertussis in Chile are immediately investigated and treated with chemoprophylaxis and are typically confirmed by PCR in reference or private laboratories; however, culture and serology may also be used.

### Colombia

The National Institute of Health of Colombia undertakes both passive and active surveillance of pertussis. Laboratory testing (culture and PCR) is performed only for hospitalized patients and as part of active surveillance.

### Costa Rica

rt-PCR testing of samples from clinically suspected cases of pertussis is performed at the national reference laboratory (INCIENSA); at the Hospital Nacional de Niños, the only national pediatric tertiary referral center in Costa Rica; and in private hospitals.

### Mexico

A sentinel network is used to actively surveil pertussis in Mexico [26]. Samples from suspected clinical cases are collected at first-level health units and sent to state-level public health laboratories for analysis by rt-PCR and/or culture. Samples identified as positive at the state level are then dispatched to the national reference laboratory (Institute of Epidemiological Diagnosis and Reference [InDRE]), where the diagnosis is confirmed via rt-PCR and the bacterium is serotyped. InDRE also performs serologic testing for antibodies against pertussis toxoid.

### Paraguay

In Paraguay, cases of pertussis must be reported to the national surveillance system (General Directorate of Health Surveillance). Suspected clinical cases are confirmed via PCR or culture.

### Peru

In Peru, national disease surveillance falls under the remit of the Ministry of Health. Samples from suspected clinical cases are analyzed centrally by the National Institute of Health and confirmed via PCR and/or culture.

### Uruguay

A sentinel surveillance system for pertussis is in place in Uruguay. All suspected clinical cases must be reported to the Department of Health Surveillance (DEVISA) and are tested using PCR, culture, or serology.

### Venezuela

Pertussis surveillance in Venezuela is undertaken by the Epidemiological Surveillance Directorate and the Expanded Program on Immunization, both of which fall under the auspices of the Ministry of Health. Clinical specimens are tested via PCR, culture, and direct fluorescent antibody testing.

## EPIDEMIOLOGY

### Argentina

Argentina experienced outbreaks of pertussis in 2011–2012 and 2016 [20], with incidences of 69 and 38.6 cases per million, respectively [31]. The incidence rate declined to 19.6–21.8 per million between 2017 and 2019, with further declines observed during the COVID-19 pandemic (Table 2). Of the 196 cases of pertussis reported in 2022, 65% occurred in infants [32] and 5% occurred in those aged ≥20 years [33].

**Table 2.** Burden of Pertussis by Year in Select Latin American Countries

Country	2017	2018	2019	2020	2021	2022	2023
No. of reported cases							
Argentina	864	900	975	162	174	196	605
Brazil	1804	2232	1423	229	143	246	210
Chile	849	682	350	62	29	0	424
Colombia	330	416	347	68	75	111	54
Costa Rica	58	36	51	10	2	5	22
Mexico	827	783	874	251	22	69	188
Paraguay	44	53	26	15	5	4	6
Peru	611	483	414	47	35	4	132
Uruguay	293	187	69	22	44	6	24
Venezuela^a^	21	…	144	217	1	0	0
Incidence rate per million							
Argentina	19.6	20.3	21.8	3.6	3.8	4.3	13.2
Brazil	8.7	10.6	6.7	1.1	0.7	1.1	1.0
Chile	46.2	36.5	18.4	3.2	1.5	0	21.6
Colombia	6.8	8.4	6.9	1.3	1.5	2.1	1.0
Costa Rica	11.6	7.1	10.0	2.0	0.4	1.0	4.2
Mexico	6.7	6.3	7.0	2.0	0.2	0.5	1.5
Paraguay	6.9	8.2	4.0	2.3	0.7	0.6	0.9
Peru	19.3	15.0	12.6	1.4	1.0	0.1	3.8
Uruguay	85.6	54.6	20.1	6.4	12.8	1.8	7.0
Venezuela^a^	0.7	…	4.8	7.5	0	0	0

Data per the World Health Organization. Available at: https://immunizationdata.who.int/global/wiise-detail-page/pertussis-reported-cases-and-incidence.

^a^Epidemiological data have not been consistently reported by the Venezuelan Ministry of Health since 2017.

### Brazil

Between the 2017 GPI meeting and the COVID-19 pandemic, the incidence rate of pertussis in Brazil ranged from 6.7 to 10.6 cases per million (Table 2). Since 2020, the rate has hovered around 1 case per million. However, these national estimates obscure state-level differences in disease burden. Of the approximately 240 cases of pertussis reported in 2022, 56% occurred in those aged <1 year, and 2.5% occurred in those aged ≥20 years [34]. According to the Pan American Health Organization, the number of suspected cases of pertussis in Brazil increased from 973 in 2023 to 1465 in 2024 [6].

### Chile

Chile experienced a pertussis outbreak in 2012 (incidence rate of 332.3 cases per million) [31]. Disease burden has since declined, with a low of 1.5 cases per million reported in 2021 (Table 2). However, in 2023, the rate increased to 21.6 per million.

### Colombia

Colombia experienced an outbreak of pertussis in 2013, with nearly 14 000 cases reported and an incidence rate of 295.9 per million [31]. Since then, the incidence rate has steadily declined, reaching an all-time low of 1 case per million in 2023 (Table 2). However, an outbreak of pertussis, which resulted in 10 deaths, was reported in the state of La Guajira in March 2022 [35]. Of the 62 confirmed cases, 26% occurred in infants, 48% in those aged 1–4 years, 15% in those aged 5–9 years, 10% in those aged 10–19 years, and 2% in those aged ≥20 years. Most cases were observed in individuals who were unvaccinated or undervaccinated.

### Costa Rica

Costa Rica experienced an epidemic of pertussis beginning in 2006 [36]. In 2007 and 2008, the incidence rate was approximately 450 cases per million, with >2000 cases reported annually [31]. During this outbreak, the greatest number of hospitalizations and deaths occurred in infants [36]. In response, the postpartum vaccination of mothers with a tetanus-diphtheria-acellular pertussis vaccine (Tdap) was introduced nationwide in 2007.

Between 2017 and 2019, the incidence rate of pertussis ranged from 7.1–11.6 cases per million, declining further to 0.4–2.0 cases per million during the pandemic (Table 2) [31]. During 2018–2021, there were 92 confirmed cases of pertussis in Costa Rica, with 59% occurring in those aged <6 months, 13% in those aged 6–11 months, 17% in those aged 1–4 years, and 11% in those aged ≥5 years [37].

### Mexico

Since 2017, the incidence rate of pertussis in Mexico has been ≤7 cases per million (Table 2) [31]. Based on data collected over 2018 and 2019, the greatest number of cases was reported in children aged 10–14 years and adults aged 20–29 years [38]. This is unique relative to other countries in Latin America, where most cases are observed in infants. In 2023, a total of 98 cases of confirmed pertussis and 950 cases of pertussis-like syndrome were reported. By week 40 of 2024, there were 332 cases of pertussis and 1289 cases of pertussis-like syndrome—a >200% increase relative to the whole of 2023 [39].

### Paraguay

Between 2017 and 2019, the incidence of pertussis in Paraguay ranged from 4.0 to 8.2 cases per million (Table 2) [31]. Since 2021, the rate has been <1 case per million.

### Peru

Between 2014 and 2016, the incidence of pertussis in Peru was <10 cases per million, with <250 cases reported annually [31]. More than 600 cases were documented in 2017, and between 2017 and 2019, the rate of pertussis was 12.6–19.3 cases per million (Table 2). Over 2018–2019, the majority of cases occurred in infants [27]. The burden of pertussis declined sharply during the COVID-19 pandemic (0.1–1.4 cases per million) and increased slightly to 3.8 cases per million in 2023 [31]. Twenty-four cases of pertussis were reported between epidemiological week 1 and week 22 of 2024, representing a nearly 5-fold increase relative to the same period in 2023 [6].

### Uruguay

In Uruguay, pertussis follows an endemic-epidemic cycle, with outbreaks generally seen every 3–5 years. The last pertussis outbreak started in 2015 [18], with a peak incidence of 228.3 cases per million [31]. By 2017, the epidemic had resolved, with declines seen each year between 2017 and 2020 (Table 2). However, there was a 2-fold increase in incidence rate between 2020 and 2021 (from 6.4 to 12.8 cases per million). Following a low of 1.8 cases per million in 2022, an uptick was observed in 2023.

### Venezuela

Due to political and socioeconomic unrest [40], disease surveillance in Venezuela has become compromised, such that the Ministry of Health has not consistently released its weekly epidemiological reports since 2017 [41].

## VACCINATION POLICIES

There is no universal vaccination scheme for pertussis, nor is there uniformity in the type of vaccine used. Instead, each country, based on its cultural traditions and socioeconomic conditions, has implemented vaccination strategies that align with recommendations from international organizations such as the World Health Organization (WHO). These recommendations have evolved in response to growing knowledge about the immunity induced by currently available vaccines, including whole-cell pertussis vaccines (wP)—the first generation of vaccines, which are more reactogenic but provide longer-lasting immunity [42, 43]—and acellular pertussis vaccines (aP)—the second generation, which are safer but induce shorter-lasting immunity [43–45]. In its 2015 position paper, the WHO stated that “the switch from wP to aP for primary infant immunization was proposed as at least partially responsible for that resurgence” [46]. The WHO further recommended that the transition to aP vaccines should only be considered if national immunization schedules can ensure the administration of multiple doses, including several boosters. Countries currently using aP vaccines may continue to do so but should evaluate the need for additional booster doses and implement strategies to prevent early childhood mortality in the event of a pertussis resurgence [46]. This section is followed by an analysis of vaccination policies across various Latin American countries, highlighting the diversity in approaches and their alignment with WHO guidelines.

### Argentina

Under the National Immunization Program (NIP), Argentinian infants receive 3 doses of a pentavalent vaccine protecting against diphtheria-tetanus-pertussis (DTP; whole cell [DTwP]), hepatitis B virus (HBV), and *Haemophilus influenzae* type b (Hib) at 2, 4, and 6 months (Table 3). In terms of boosting, DTwP-Hib is administered at 15–18 months, DTwP at 5 years, and Tdap at 11 years. Acellular vaccine formulations are generally reserved for premature infants in the public sector but are available for purchase in the private sector.

**Table 3.** Overview of Pertussis Vaccine Schedules and Coverage in Select Latin American Countries

Schedule		Argentina	Brazil	Chile	Colombia	Costa Rica	Mexico	Paraguay	Peru	Uruguay	Venezuela
Primary series		2, 4, and 6 mo									
Coverage^a^	DTP1	87%	84%	99%	91%	98%	93%	82%	93%	99%	73%
	DTP3	81%	77%	96%	87%	95%	83%	69%	82%	94%	56%
Booster^b^ dose 1		15–18 mo	15 mo	18 mo	18 mo	15 mo	18 mo	18 mo	18 mo	15 mo	15–18 mo
Booster^b^ dose 2		5 y	4 y	6 y	5 y	4 y	4 y	4 y	4 y	5 y	3–6 y
Booster^b^ dose 3		11 y	…	12–13 y	…	…	…	…	…	11 y	11–12 y
Adults		HCP^c^	HCP only (every 10 y)	…	…	…	…	…	…	HCP^c^, Cocoon^d^	…
Pregnant persons		Yes	Yes	Yes	Yes	Yes	Yes	No^e^	Yes	Yes	No^e^

Abbreviations: DTP1, first dose of diphtheria-tetanus-pertussis vaccine; DTP3, third dose of diphtheria-tetanus-pertussis vaccine; HCP, healthcare provider.

^a^Coverage estimates (2022) derive from World Health Organization reports available at https://immunizationdata.who.int/dashboard.

^b^Recommended age at vaccination.

^c^Only HCPs who come into contact with those <12 months of age (ie, infants).

^d^Parents and/or caregivers of newborns.

^e^Recommendations are in place in the private sector.

Since 2012, Tdap has been recommended for pregnant persons with a gestational age of ≥20 weeks. In an analysis performed at the Ricardo Gutiérrez Children's Hospital in Buenos Aires, hospitalizations due to pertussis decreased from 22.3 per 10 000 discharges in the pre-ViP era (2003–2011) to 11.6 per 10 000 discharges during the post-ViP era (2013–2016), a reduction of 48% [47]. As only 61% of pregnant persons in Argentina received Tdap in 2023, the VCR in this target group remains suboptimal [33]. Since 2009, there has been a recommendation in place for healthcare providers (HCPs) who encounter children aged <1 year to receive Tdap.

### Brazil

The Brazilian Public Health System endorses immunization with DTwP-HBV-Hib at 2, 4, and 6 months of age and booster doses of DTwP at 15 months and 4 years of age (Table 3). However, diphtheria-tetanus-acellular pertussis (DTaP) is available at the Reference Centers for Special Immunobiologicals for children aged <7 years who are at an increased risk of developing or who previously developed serious adverse reactions to DTwP [25].

Tdap is available without cost to HCPs aged 20–59 years and to pregnant persons. Unfortunately, only 47% of pregnant persons received Tdap in 2022 [48]. Tdap is also available for purchase in the private sector. The Brazilian Immunization Society recommends universal adolescent (10–19 years) and adult (≥20 years) immunization with Tdap [49].

### Chile

In 2018, a hexavalent vaccine, DTaP-HBV-Hib-inactivated polio virus (IPV), was introduced to the NIP in Chile, with doses for the primary series administered at 2, 4, and 6 months of age and a booster dose administered at 18 months of age (Table 3). Tdap boosters are recommended for those aged 6 years and 12–13 years and for those who are pregnant (≥28 weeks of gestation). In 2022, the VCR among pregnant persons in Chile was 69% [50].

### Colombia

The national vaccination schedule for pertussis in Colombia involves 3 doses of DTwP-HBV-Hib administered at 2, 4, and 6 months of age and 2 booster doses of DTwP administered at 18 months and 5 years (Table 3). Since 2014, Tdap has been recommended nationally for pregnant persons with a gestational age of ≥26 weeks. However, coverage is estimated at only 39% [51]. In the private sector, Tdap is available to those aged 11–17 years.

### Costa Rica

Under the NIP, infants in Costa Rica receive DTaP-Hib-IPV at 2, 4, 6, and 15 months of age and DTaP-IPV at 4 years of age (Table 3). The switch from wP to aP vaccines for the primary immunization series and booster occurred in 2010. In 2011, ViP was introduced [36], with a coverage rate of 96% in 2024 [52]. Between 2012 and 2018 (ie, following the introduction of ViP), there have been only 4 deaths due to pertussis: 3 in infants aged <1 month and 1 in an individual aged ≥12 months [36].

### Mexico

Under the Universal Vaccine Program, DTaP-Hib-IPV is administered at 2, 4, 6, and 18 months followed by DTwP or DTaP at 4 years (Table 3). The switch from wP to aP vaccines for the primary immunization series and first booster occurred in 2007. ViP was introduced in Mexico in 2013. A comparison of the pre-ViP (2010–2012) and post-ViP (2014–2018) periods revealed that pertussis incidence and pertussis-related hospitalizations and deaths among infants aged <2 months decreased by 50%, 70%, and 82%, respectively. In a national seroepidemiological surveillance study performed in 2022, 58% of women of childbearing age (29–49 years) were estimated to have received Tdap [53]. The results of this study show that a considerable proportion of women of childbearing age are not protected against pertussis [53].

### Paraguay

Infants in Paraguay are immunized with DTaP-Hib-IPV at 2, 4, and 6 months of age and with DTaP at 18 months and 4 years of age (Table 3). The switch from wP to aP vaccines for the primary immunization series and boosters occurred in 2012. Although there is no formal national endorsement, the Paraguayan Society of Infectious Diseases (SPI), a national medical society, recommends that pregnant persons with a gestational age of ≥20 weeks receive Tdap. SPI also advises that all individuals aged ≥19 years receive tetanus-diphtheria vaccine (Td).

### Peru

Peruvian children receive DTwP-HBV-Hib at 2, 4, and 6 months of age and are boosted with DTwP at 18 months and 4 years (Table 3). In 2019, ViP was introduced in Peru, with Tdap administered between 27 and 36 weeks of gestation. The estimated VCR among pregnant persons in 2022 was 56% [54]. In a case-control study performed in Peru over 2019–2022, the infants of mothers who received Tdap during their third trimester had an 81% lower risk of pertussis than those born to nonvaccinated mothers [55].

### Uruguay

Under the NIP in Uruguay, DTwP-HBV-Hib is administered to infants aged 2, 4, 6, and 15 months; DTwP to those aged 5 years; and Tdap to those aged 11 years (Table 3). Universal ViP has been recommended since 2012, but Tdap only became considered mandatory and freely available to pregnant persons in 2015. Although available to those with a gestational age of ≥20 weeks, DEVISA prefers that pregnant persons receive Tdap at 28–36 weeks of gestation. In a single-center survey performed in 2019, an estimated 55% of pregnant persons received Tdap, with 18% refusing Tdap outright [56].

Uruguay was the only country represented at the 2023 GPI meeting to have implemented a cocooning strategy. Following the birth of a child, parents or up to 4 caregivers can receive Tdap free of charge. Since 2015, DEVISA has recommended that HCPs who come into contact with children aged <12 months receive Tdap.

### Venezuela

Per the Venezuelan Society of Childcare and Pediatrics, children should receive DTwP or DTaP at 2, 4, and 6 months of age; DTaP at 3–6 years; and Tdap at 11–12 years (Table 3). Given the country's current situation, it is not surprising that VCRs for the first dose of DTP (DTP1) and the third dose of DTP (DTP3) are low (73% and 56%, respectively), increasing outbreak potential. In fact, after having been eradicated in 1992, diphtheria resurfaced in Venezuela in 2016 [57]. Increasing coverage rates for the primary vaccination series should be the top priority for pertussis control efforts in Venezuela.

Per the Society of Gynecology and Obstetrics of Venezuela, pregnant persons should receive Tdap at >20 weeks of gestation. If not, it is mandatory that they receive Tdap in the immediate postpartum period. Pregnant persons are vaccinated in private practice by their obstetrician/gynecologist.

## DISCUSSION

### Surveillance

Although pertussis is a notifiable disease in all 10 Latin American countries represented at the 2023 GPI meeting, there are challenges associated with robust surveillance in the region. These include barriers to case notification, poor awareness and underrecognition of pertussis in older age groups, limited laboratory capacity and supplies, variations in testing strategies, and lack of a standardized case definition (Table 4) [58–60]. Potential ways to overcome or mitigate these challenges include the development of reporting platforms that are easier to navigate, such as the inclusion of drop-down menus and mobile device–friendly applications; strengthening existing laboratory networks, including adapting test streams developed in response to the COVID-19 pandemic [61, 62]; and introducing a single case definition that reflects the disparate clinical presentation of pertussis in younger versus older individuals and that includes the use of anti–pertussis toxin immunoglobulin G antibody thresholds for case positivity [60].

**Table 4.** Challenges and Potential Solutions to Pertussis Surveillance in Latin America

Challenge	Potential Solution
Barriers to case notification	Develop platforms that are easier to navigate and that are less time-consuming (eg, case reports with drop-down menus, mobile device–friendly options) Incentivize HCPs to complete case reports (eg, reimbursement for completion, penalties for noncompliance)
Poor awareness of pertussis	Educate HCPs on the signs and symptoms of pertussis in adults (and adolescents), so that the appropriate diagnostic tests can be run
Underrecognition of pertussis	
Limited laboratory capacity/supplies and variations in testing strategies	Emphasize the importance of accurate diagnoses with decision-makers, so that they can assist with securing resources for improved testing Strengthen existing laboratory networks (eg, introduce multiplex testing) Implement point-of-care testing (eg, rapid antigen tests) to avoid waiting for results from reference laboratories Harmonize anti-PT IgG antibody thresholds for determining case-positivity via serology Train HCPs on proper swabbing techniques
Lack of a standardized case definition	Consider a case definition that includes all laboratory-confirmed cases, not just those diagnosed via PCR Allow for suspected cases to be considered positive through epidemiological linkage and/or the satisfaction of clinical criteria Tailor the definition to reflect the disparate clinical presentation of pertussis in infants/children vs adolescents/adults

Abbreviations: HCP, healthcare provider; IgG, immunoglobulin G; PCR, polymerase chain reaction; PT, pertussis toxin.

Additional benefit lies in educating the public and HCPs on pertussis. The former can focus on “community,” namely how vaccination protects not only the recipient but those who are vulnerable (eg, unimmunized neonates, partially immunized infants). Through its distinct clinical presentation, HCPs should be educated on the signs and symptoms of pertussis in adults. This is important because underrecognition of pertussis as a potential cause of cough in adults [63–65] leads to undertesting and underdiagnosis [66, 67], which, in turn, results in underreporting [13, 66]. Indeed, a Canadian-based modeling analysis suggested that there could have been as many as 33 300 unreported cases of pertussis for every reported case among Canadians aged 20–64 years [66].

The meeting attendees were unanimous in their belief that HCPs who provide care to adults are less aware of pertussis than are pediatricians. Thus, the GPI endorses the education of all HCPs (eg, general practitioners, obstetricians/gynecologists, midwives) on pertussis, not just pediatricians. Educating future HCPs at the university level and beyond in pertussis disease presentation will ultimately aid in gaining insight into the true burden of pertussis in adults.

### Impact of COVID-19

The incidence rate of pertussis across Latin America declined substantially during the COVID-19 pandemic (Table 2), a consequence of stay-at-home orders, social distancing, travel restrictions, and universal masking. The exception lay in Venezuela, where the incidence rate was 4.8 cases per million in 2019 and 7.5 cases per million in 2020 (Table 2). However, these data should be interpreted with caution, as turmoil in the country has caused disruptions to healthcare services and lapses in disease reporting. Thus, the true impact of COVID-19 on the burden of pertussis in Venezuela cannot be accurately assessed. Epidemiological data from countries like Argentina, Chile, and Costa Rica indicate a gradual return to prepandemic levels of pertussis. Potential factors contributing to this reemergence include pandemic-related lapses in pertussis vaccination, waning of natural boosting due to reduced exposure to *Bordetella pertussis* during lockdown, the lifting of social distancing measures, reduced use of face masks, and HCP shortages.

Across most Latin American countries, there was a decrease in immunization against pertussis during the height of the pandemic. For example, compared with 2019, the coverage rate for DTP1 in Paraguay was reduced by 5% in 2020 and by 13% in 2021; the corresponding declines for DTP3 were 9% and 22% [68]. In Brazil, the VCR among pregnant persons was 63% in 2019, but only 46% in 2020 and 43% in 2021 [48]. In Mexico, DTP3 coverage for the years 2021 and 2022 was 70.1% and 69.0%, respectively [69]. In contrast, vaccination coverage in Uruguay was relatively unaffected by the pandemic, with DTP1 rates of 100% in 2019 and 98% in both 2020 and 2021; as to DTP3, the VCR was 94% in 2019, 92% in 2020, and 91% in 2021 [70]. Similarly, the VCR rate for DTP3 in Costa Rica in 2019, 2020, and 2021 was 95%, 97%, and 93%, respectively [70]. COVID-19–related factors that likely contributed to lower VCRs include limited healthcare access and staff availability, cancellation of immunization visits due to fear of exposure to severe acute respiratory syndrome coronavirus 2, and vaccine shortages. Catch-up vaccination campaigns against pertussis are needed to minimize outbreak risk.

### Vaccination

In all 10 Latin American countries, infants are immunized with either a wP- or aP-containing vaccine at 2, 4, and 6 months of age, with booster doses administered at 15–18 months and 3–6 years (Table 3). However, only the national programs of Argentina, Chile, Uruguay, and Venezuela endorse a third booster dose (Tdap) at 11–13 years. VCRs for the primary vaccination series vary across countries, ranging from 73% to 99% for DTP1 and 56% to 96% for DTP3 (Table 3) [71].

Due to the passive transfer of maternal antibodies [72–74], ViP is effective in protecting infants aged <2 months from pertussis [75]. The government health agencies of all represented Latin American countries advise or mandate ViP, except for Paraguay and Venezuela (Table 3). However, in Paraguay, an influential professional society (SPI) recommends that Tdap be administered during each pregnancy. Among the 8 countries with national-level ViP policies, the VCRs for pregnant persons remain suboptimal [33, 37, 48, 50, 51, 54, 56].

Major challenges to the uptake of Tdap among pregnant persons in Latin America include poor awareness of pertussis among HCPs providing prenatal care, misperception of vaccines, and lack of strong provider recommendations (Table 5). Overall, education is key to improving VCRs among those who are pregnant. This education should be targeted to obstetricians/gynecologists, midwives, and pregnant persons and should illustrate how ViP (1) is effective in preventing pertussis in infants before the start of their primary vaccination series at 2 months [75, 76], (2) reduces the number of infant hospitalizations and deaths from pertussis [47, 77], and (3) does not affect pregnancy outcomes in either mothers or infants [72, 74, 78]. ViP guidelines should be clear and easily accessible to HCPs, who should communicate on the effectiveness and safety of vaccination. Vaccine access is also a hindrance to high VCRs for pregnant persons and could be improved by initiating vaccination campaigns in primary care centers and hospitals and by providing home health visits during pregnancy. Switching from Td, which is often routinely administered during pregnancy, to Tdap would also broaden protection against pertussis. When deciding whether to introduce ViP, policy makers need to consider several factors, including the timing of vaccination (second vs third trimester) and the need to vaccinate in each pregnancy.

**Table 5.** Challenges and Potential Solutions to the Uptake of Tetanus-Diphtheria-Acellular Pertussis Vaccine Among Pregnant Persons in Latin America

Challenge	Proposed Solution
Poor awareness of pertussis among pregnant persons and HCPs providing prenatal care	Undertake public health campaigns that: Communicate on the dangers of pertussis (eg, personal stories) and the safety/effectiveness of Tdap Emphasize how ViP protects infants when they are most vulnerable (ie, the first 2–3 mo of life) Engage with medical societies whose members specialize in prenatal care (ie, obstetricians/gynecologists, midwives) on the importance of ViP Incorporate/broaden vaccine education into medical school curricula Ensure vaccination guidelines are easily accessible to HCPs providing prenatal care Develop clear and simple talking points on ViP that HCPs could communicate to their patients during routine prenatal visits
Lack of strong provider recommendations	
Misunderstanding of vaccines	
Vaccine hesitancy	Develop print and television ads that emphasize the dangers of pertussis and the safety/effectiveness of Tdap Use social media to counter vaccine misinformation
Logistics	Employ vaccine campaigns in urban and suburban areas Deploy mobile vaccine units to remote areas Leverage cold chain and storage solutions employed during disaster situations

Abbreviations: HCP, healthcare provider; Tdap, tetanus-diphtheria-acellular pertussis; ViP, vaccination in pregnancy.

Adult vaccination with Tdap is not universally recommended in any of the 10 Latin American countries represented at the GPI meeting. However, specific segments of the adult population are advised to receive Tdap in Argentina (HCPs who come into contact with infants), Brazil (HCPs), and Uruguay (HCPs who come into contact with infants and parents/caregivers of newborns) (Table 3). Factors that have hindered universal immunization with Tdap among adults are the misperception that childhood vaccines provide lifelong protection against pertussis; belief among HCPs, policy makers, and the public that pertussis is a childhood disease; lack of knowledge within the community about the ability of adults to transmit *B pertussis* to infants; and cost (Table 6). As a first step, the misperception that childhood vaccination provides lifelong protection against pertussis must be corrected. Thus, while new vaccines that induce a stronger memory response and confer longer-term immunity are being sought, there is a need to better educate on waning immunity in medical school and to initiate public health campaigns that showcase how vaccination is part of healthy aging (ie, “vaccines are not just for kids”). Hopefully, this 2-pronged approach will engender conversations between adults and their HCPs, such that patients proactively ask for and physicians offer Tdap.

**Table 6.** Challenges and Potential Solutions to the Universal Vaccination of Adults With Tetanus-Diphtheria-Acellular Pertussis in Latin America

Challenge	Proposed Solution
Misunderstanding of vaccines	Correct the misperception among the public and HCPs that childhood vaccines provide lifelong protection against pertussis Ensure that medical school curricula address waning immunity Initiate public health campaigns that showcase how vaccination is part of healthy aging (ie, “vaccines are not just for kids”) Illustrate the effectiveness and utility of vaccines by showing how they have been used to control recent pertussis outbreaks
Belief that pertussis is a childhood disease	Undertake local/regional epidemiological studies to characterize the burden of asymptomatic infection and overt disease in adults Educate HCPs on the signs and symptoms of pertussis in adults, so that the appropriate diagnostic tests can be run Advise HCPs of how serology may be more appropriate for the diagnosis of pertussis in adults than PCR or culture Enhance disease surveillance in older individuals (see Table 4)
Lack of community-level knowledge about the ability of adults to transmit *Bordetella pertussis* to infants	Undertake public health campaigns that emphasize how vaccination protects not only the recipient (directly) but those who are too young to be vaccinated (indirectly)
Cost	Perform local/regional health economic studies to evaluate the cost-effectiveness of introducing universal adult immunization with Tdap Consider the direct costs associated with adults who develop severe pertussis and the indirect costs associated with infants who contract *B pertussis* from adults

Abbreviations: HCP, healthcare provider; PCR, polymerase chain reaction; Tdap, tetanus-diphtheria-acellular pertussis.

It is important to note that pertussis can also be a notable disease for adults, particularly for those with asthma and chronic obstructive pulmonary disease, who are at higher risk for complications [79, 80]. Robust epidemiological data are the only solution to rectifying the erroneous belief that pertussis exclusively affects children. An analysis of 5 Latin American countries estimated that the number of pertussis cases among adults aged ≥50 years is approximately 100 times higher than that suggested by passive surveillance [58]. Consequently, both the burden of asymptomatic infection and overt disease in adults should be quantified through formal study. However, to accurately capture the true burden of disease, HCPs must recognize the signs and symptoms of pertussis in adults, which could be achieved through webinars, continuing education courses, and/or the distribution of educational signs and pamphlets. Given that adults may wait >1 week to seek medical attention for a persistent cough and the fact that antibiotics may be more liberally prescribed in some countries than in others, single-sample serological testing against predefined positivity levels is likely the most appropriate diagnostic approach to detecting a recent infection with *B pertussis* in this age group.

Vaccination of the adult population with Tdap will be costly, irrespective of whether those costs are borne by the recipient or the government. To determine whether such a broad-reaching vaccination policy is cost effective, country-level health economic evaluations are needed. To maximize their real-world value, these evaluations require understanding of the true scope of adult disease (ie, accurate epidemiological data) and consideration of the direct and indirect costs of infection with *B pertussis*. However, indirect costs, such as those associated with infants who contract *B pertussis* from an adult and the resulting medical interventions (eg, antibiotics, hospitalization) and outcomes (eg, death, long-term sequelae, resolution), may be difficult to quantify.

## CONCLUSIONS

Pertussis continues to pose a considerable public health challenge in Latin America, particularly among infants, and efforts to increase vaccination coverage and strengthen surveillance are crucial to reducing the burden of disease in this region. The childhood immunization schedules for pertussis in the region are robust, with ≥2 booster doses following the primary vaccination series in all represented countries. As to the primary vaccination series, there is a need to maintain high coverage with DTP for countries whose rates exceed 90% (eg, Chile, Costa Rica, Uruguay), to bolster coverage for countries whose rates have historically been low (eg, Venezuela), and to restore coverage for countries whose rates were adversely affected by the COVID-19 pandemic (eg, Paraguay). Although there are national-level recommendations for ViP in most countries represented at the 2023 GPI meeting, VCRs among pregnant persons are universally subpar. To remedy this, HCPs providing antenatal care and pregnant persons should be educated on (1) how mothers can serve as asymptomatic carriers of *B pertussis* and (2) the increased vulnerability of neonates to pertussis in the first 2–3 months of life. Because pertussis can affect persons of any age, the GPI recommends boosters for adolescents and adults [13]. However, to justify the expansion of Tdap recommendations from a scientific, political, and financial perspective, accurate epidemiological data are needed. To augment the passive surveillance of pertussis, HCPs caring for adults in the region need to be educated on the signs and symptoms of pertussis in older individuals, as the clinical presentation is often different from that of infants and children. Ultimately, the burden of pertussis in Latin America can be reduced by increasing vaccine uptake among pregnant persons and HCPs and, if supported by the data, by adopting the universal vaccination of adults with Tdap.

## Notes

***Acknowledgments*.** Medical writing and editorial support for the development of this manuscript, under the direction of the authors, was provided by Tiffany DeSimone and Katie Holmes of Ashfield MedComms, an Inizio company, and was funded by Sanofi.

***Author contributions*.** All authors were responsible for idea generation, critical review, and input on the manuscript drafts, as well as approval of the final draft for submission.

***Disclaimer.*** Sanofi had no input or access to the content of this manuscript.

***Financial support.*** The Global Pertussis Initiative is supported by an unrestricted educational graft from Sanofi.

## References

[1] European Centre for Disease Prevention and Control . Increase of pertussis cases in the EU/EEA. 2024. Available at: https://www.ecdc.europa.eu/en/publications-data/increase-pertussis-cases-eueea. Accessed October 10, 2024.

[2] Wan M, Zhang G, Yi H. Unraveling the resurgence of pertussis: insights into epidemiology and global health strategies. Pulmonology 2024; 30:503–5. 38755090 10.1016/j.pulmoe.2024.04.009

[3] Liu Y, Ye Q. Resurgence and the shift in the age of peak onset of pertussis in southern China. J Infect 2024; 89:106194. 38830410 10.1016/j.jinf.2024.106194

[4] Hu Y, Shi W, Meng Q, et al Detection of *Bordetella* spp. in children with pertussis-like illness from 2018 to 2024 in China. J Infect 2024; 89:106222. 39002934 10.1016/j.jinf.2024.106222

[5] Khalil A, Samara A, Campbell H, Ladhani SN, Amirthalingam G. Recent increase in infant pertussis cases in Europe and the critical importance of antenatal immunizations: we must do better … now. Int J Infect Dis 2024; 146:107148. 38960028 10.1016/j.ijid.2024.107148

[6] Pan American Health Organization . Epidemiological alert—pertussis (whooping cough) in the region of the Americas. 2024. Available at: https://www.paho.org/en/documents/epidemiological-alert-pertussis-whooping-cough-region-americas-22-july-2024. Accessed October 10, 2024.

[7] Gabutti G, Rota MC. Pertussis: a review of disease epidemiology worldwide and in Italy. Int J Environ Res Public Health 2012; 9:4626–38. 23330226 10.3390/ijerph9124626 PMC3546780

[8] Kaczmarek MC, Ware RS, McEniery JA, Coulthard MG, Lambert SB. Epidemiology of pertussis-related paediatric intensive care unit (ICU) admissions in Australia, 1997–2013: an observational study. BMJ Open 2016; 6:e010386. 10.1136/bmjopen-2015-010386 PMC4823423 27053270

[9] Marshall H, Clarke M, Rasiah K, et al Predictors of disease severity in children hospitalized for pertussis during an epidemic. Pediatr Infect Dis J 2015; 34:339–45. 25260040 10.1097/INF.0000000000000577

[10] World Health Organization . Pertussis vaccines: WHO position paper—August 2015. 2015. Available at: https://www.who.int/publications/i/item/WHO-WER9035. Accessed November 1, 2024.

[11] Yeung KHT, Duclos P, Nelson EAS, Hutubessy RCW. An update of the global burden of pertussis in children younger than 5 years: a modelling study. Lancet Infect Dis 2017; 17:974–80. 28623146 10.1016/S1473-3099(17)30390-0

[12] Harrington L, Aris E, Bhavsar A, et al Burden of pertussis in adults aged 50 years and older: a retrospective database study in England. Infect Dis Ther 2023; 12:1103–18. 36966230 10.1007/s40121-023-00774-5 PMC10147870

[13] Forsyth KD, Campins-Marti M, Caro J, et al New pertussis vaccination strategies beyond infancy: recommendations by the Global Pertussis Initiative. Clin Infect Dis 2004; 39:1802–9. 15578403 10.1086/426020

[14] Centers for Disease Control and Prevention . Pertussis epidemic—Washington, 2012. MMWR Morb Mortal Wkly Rep 2012; 61:517–22. 22810264

[15] Forsyth KD, Wirsing von Konig CH, Tan T, Caro J, Plotkin S. Prevention of pertussis: recommendations derived from the second Global Pertussis Initiative roundtable meeting. Vaccine 2007; 25:2634–42. 17280745 10.1016/j.vaccine.2006.12.017

[16] Forsyth K, Plotkin S, Tan T, Wirsing von König CH. Strategies to decrease pertussis transmission to infants. Pediatrics 2015; 135:e1475–82. 25963002 10.1542/peds.2014-3925

[17] Abu-Raya B, Forsyth K, Halperin SA, et al Vaccination in pregnancy against pertussis: a consensus statement on behalf of the Global Pertussis Initiative. Vaccines (Basel) 2022; 10:1990. 36560400 10.3390/vaccines10121990 PMC9786323

[18] Hozbor D, Ulloa-Gutierrez R, Marino C, Wirsing von König CH, Tan T, Forsyth K. Pertussis in Latin America: recent epidemiological data presented at the 2017 Global Pertussis Initiative meeting. Vaccine 2019; 37:5414–21. 31331774 10.1016/j.vaccine.2019.07.007

[19] Pinell-McNamara VA, Acosta AM, Pedreira MC, et al Expanding pertussis epidemiology in 6 Latin America countries through the Latin American Pertussis Project. Emerg Infect Dis 2017; 23:S94–100. 29155677 10.3201/eid2313.170457 PMC5711316

[20] Fabricius G, Martin Aispuro P, Bergero P, Bottero D, Gabrielli M, Hozbor D. Pertussis epidemiology in Argentina: TRENDS after the introduction of maternal immunisation. Epidemiol Infect 2018; 146:858–66. 29655385 10.1017/S0950268818000808 PMC9184940

[21] Gentile A, Bricks L, Ávila-Agüero ML, et al Pertussis in Latin America and the Hispanic Caribbean: a systematic review. Expert Rev Vaccines 2019; 18:829–45. 31317794 10.1080/14760584.2019.1643241

[22] Ulloa-Gutierrez R, Hozbor D, Avila-Aguero ML, et al The Global Pertussis Initiative: meeting report from the regional Latin America meeting, Costa Rica, 5–6 December, 2008. Hum Vaccin 2010; 6:876–80. 20980794 10.4161/hv.6.11.13077

[23] Ministeria da Saude. Coqueluche. Available at: https://www.gov.br/saude/pt-br/assuntos/saude-de-a-a-z/c/coqueluche. Accessed October 10, 2024.

[24] Ministerio de Salud, Costa Rica . National pertussis surveillance protocol of 2009. Addendum March 8, 2019 [in Spanish]. 2019. Available at: https://www.ministeriodesalud.go.cr/index.php/biblioteca-de-archivos-left/documentos-ministerio-de-salud/vigilancia-de-la-salud/normas-protocolos-guias-y-lineamientos/1819-adenda-protocolo-tosferina/file#:∼:text=Adenda 8 Marzo 2019 DVS,Tosferina Costa Rica del 2009.&text=El caso sospechoso%20es una,una duraci%C3%B3n %E2%89%A5 2 semanas.&text=Ni%C3%B1os menores de un a%C3%B1o con episodios de%20apnea de cualquier duraci%C3%B3n. Accessed November 1, 2024.

[25] Health surveillance guide [in Portuguese]. 2022. Available at: https://www.vs.saude.ms.gov.br/wp-content/uploads/2023/02/Guia-de-Vigilancia-em-Saude-5a-ed-atual.pdf. Accessed November 1, 2024.

[26] Guidelines for laboratory surveillance of pertussis and pertussis-like syndrome. 2017. Available at: https://www.gob.mx/cms/uploads/attachment/file/487572/LVL_Tosf_4T.pdf. Accessed December 1, 2024.

[27] Epidemiological surveillance, prevention and control of pertussis 2019 SE 47* 2024. Available at: https://www.dge.gob.pe/portal/docs/tools/teleconferencia/2019/SE482019/05.pdf. Accessed December 15, 2024.

[28] Latin American Pertussis Project Workshop: results of epidemiological surveillance of whooping cough in Chile and the Latin American Project of Pertussis (LAPP) [in Spanish]. 2019. Available at: www.sabin.org/app/uploads/2022/07/Ivan-Rios.pdf.pdf. Accessed December 10, 2024.

[29] Pertussis public health surveillance protocol in Colombia. 2024. Available at: https://www.ins.gov.co/buscador-eventos/Lineamientos/Pro_Tos%20ferina%202024.pdf. Accessed November 1, 2024

[30] Coqueluche, tos convulsa, tos ferina Paraguay. 2024. Available at: https://dgvs.mspbs.gov.py/enfermedades/coqueluche-tos-convulsa-tos-ferina/. Accessed October 12, 2024.

[31] World Health Organization . Pertussis reported cases and incidence. 2024. Available at: https://immunizationdata.who.int/global/wiise-detail-page/pertussis-reported-cases-and-incidence. Accessed October 10, 2024.

[32] Avila-Agüero ML, Ospina-Henao S, Mariño C, et al Vaccination against pertussis in Latin American preterm and low-birth weight infants: experts opinion position for a neglected childhood age group. Expert Rev Vaccines 2023; 22:1126–35. 37814599 10.1080/14760584.2023.2268712

[33] Ministerio de Salud, Argentina . National Health Surveillance System (SNVS C2–SIVILA) and SNVS2.0. Adaptation of DiCEI based on data from the National Health Surveillance System [in Spanish]. 2023. Available at: https://www.argentina.gob.ar/salud/epidemiologia. Accessed October 1, 2024.

[34] Pertussis—confirmed cases reported in the notifiable diseases information system [in Portuguese]. 2022. Available at: http://tabnet.datasus.gov.br/cgi/tabcgi.exe?sinannet/cnv/coquebr.def. Accessed October 1, 2024.

[35] Pertussis event report [in Spanish]. 2022. Available at: https://www.ins.gov.co/buscador-eventos/BoletinEpidemiologico/2022_Bolet%C3%ADn_epidemiologico_semana_39.pdf. Accessed October 1, 2024.

[36] Avila-Agüero ML, Camacho-Badilla K, Ulloa-Gutierrez R, Espinal-Tejada C, Morice-Trejos A, Cherry JD. Epidemiology of pertussis in Costa Rica and the impact of vaccination: a 58-year experience (1961–2018). Vaccine 2022; 40:223–8. 34903370 10.1016/j.vaccine.2021.11.078

[37] Costa Rica National Reference Center for Bacteriology, INCIENSA . Adapted based on data from the National Reference Center for Bacteriology [in Spanish]. 2023. Available at: https://www.inciensa.sa.cr/. Accessed October 1, 2024.

[38] Gentile Á, Torres-Torreti JP, López-López P, Ulloa-Gutierrez R. Epidemiologic changes and novelties on vaccination against *Bordetella pertussis* in Latin America. Rev Chilena Infectol 2021; 38:232–42. 34184715 10.4067/S0716-10182021000200232

[39] General Directorate of Epidemiology . Epidemiological bulletin. National Epidemiological Surveillance System [in Mexico]. 2024. Available at: https://www.gob.mx/cms/uploads/attachment/file/950839/sem40.pdf. Accessed October 10, 2024.

[40] Grillet ME, Hernández-Villena JV, Llewellyn MS, et al Venezuela's humanitarian crisis, resurgence of vector-borne diseases, and implications for spillover in the region. Lancet Infect Dis 2019; 19:e149–61. 30799251 10.1016/S1473-3099(18)30757-6

[41] Human Rights Watch . World report: Venezuela. 2023. Available at: https://www.hrw.org/world-report/2023/country-chapters/venezuela. Accessed October 1, 2024.

[42] Korkmaz HA, Aydin A, Unal B. Comparison of acellular pertussis-tetanus-diphtheria vaccines and whole-cell pertussis-tetanus-diphtheria vaccines in infancy. Paediatr Int Child Health 2014; 34:198–202. 24621240 10.1179/2046905513Y.0000000110

[43] Szwejser-Zawislak E, Wilk MM, Piszczek P, Krawczyk J, Wilczyńska D, Hozbor D. Evaluation of whole-cell and acellular pertussis vaccines in the context of long-term herd immunity. Vaccines (Basel) 2022; 11:1. 36679846 10.3390/vaccines11010001 PMC9863224

[44] Herzog C . Changing from whole-cell to acellular pertussis vaccines would trade superior tolerability for inferior protection. Expert Rev Vaccines 2015; 14:1065–72. 26098640 10.1586/14760584.2015.1059759

[45] Wilkinson K, Righolt CH, Elliott LJ, Fanella S, Mahmud SM. Pertussis vaccine effectiveness and duration of protection—a systematic review and meta-analysis. Vaccine 2021; 39:3120–30. 33934917 10.1016/j.vaccine.2021.04.032

[46] World Health Organization . Pertussis vaccines: WHO position paper, August 2015–recommendations. Vaccine 2016; 34:1423–5. 26562318 10.1016/j.vaccine.2015.10.136

[47] Gentile A, Juarez MDV, Lucion MF, et al *Bordetella pertussis* (Bp) disease: before (2003–2011) and after (2013–2016) maternal immunization strategy in a pediatric hospital. Vaccine 2018; 36:1375–80. 29429812 10.1016/j.vaccine.2018.01.091

[48] Sistema de Informação de Agravos de Notificação (SINAN). Pertussis [in Portuguese]. 2022. Available at: https://portalsinan.saude.gov.br/coqueluche. Accessed October 1, 2024.

[49] SBIm adult vaccination calendar [in Portuguese]. 2024. Available at: https://sbim.org.br/images/calendarios/calend-sbim-adulto.pdf. Accessed October 10, 2024.

[50] Ministerio de Salud de Chile . Available at: https://saludresponde.minsal.cl/wp-content/uploads/2024/12/2025.02.20_CALENDARIO-INMUNIZACIONES-2025.pdf. Accessed October 10, 2024.

[51] McDermid P, Blazek K, Mougin N, Thomson A, Seale H. Attitudes and behaviors of maternal Tdap vaccination in Panama, Peru, and Colombia: an international cross-sectional study. Vaccine 2024; 42:1698–703. 38355320 10.1016/j.vaccine.2024.01.106

[52] Epidemiological bulletin N°17 of 2024. 2024. Available at: https://www.ministeriodesalud.go.cr/index.php/biblioteca-de-archivos-left/documentos-ministerio-de-salud/material-informativo/material-publicado/boletines/boletines-vigilancia-vs-enfermedades-de-transmision-vectorial/boletines-epidemiologicos-2024/7487-boletin-epidemiologico-n-17-2/file. Accessed October 10, 2024.

[53] Mongua-Rodríguez N, Ferreira-Guerrero E, Delgado-Sánchez G, et al Vacunación en adultos y adultos mayores en México. Salud Publica Mex 2023; 65:S146–52. 38060956 10.21149/14786

[54] The vaccination campaign for pregnant women begins at the INMP [in Spanish]. 2022. Available at: https://www.inmp.gob.pe/noticia/se-inicia-la-campana-de-vacunacion-a-gestantes-en-el-inmp. Accessed October 10, 2024.

[55] Juscamayta-López E, Valdivia F, Soto MP, Horna H, Pajuelo M. Case-control study to estimate the association between Tdap vaccination during pregnancy and reduced risk of pertussis in newborn infants in Peru, 2019-2021. Open Forum Infect Dis 2023; 10:ofad325. 37469614 10.1093/ofid/ofad325 PMC10352646

[56] Guzman-Holst A, Petrozzi V, Velez C, et al Knowledge, attitudes, and practices concerning maternal immunization among pregnant/postpartum women and health care professionals in Peru. Infect Dis Ther 2023; 12:1151–73. 36966229 10.1007/s40121-023-00788-z PMC10039770

[57] Lodeiro-Colatosti A, Reischl U, Holzmann T, Hernández-Pereira CE, Rísquez A, Paniz-Mondolfi AE. Diphtheria outbreak in Amerindian communities, Wonken, Venezuela, 2016–2017. Emerg Infect Dis 2018; 24:1340–4. 29912686 10.3201/eid2407.171712 PMC6038745

[58] Guzman-Holst A, Gomez JA, Cintra O, Van Oorschot D, Jamet N, Nieto-Guevara J. Assessing the underestimation of adult pertussis disease in five Latin American countries. Infect Dis Ther 2023; 12:2791–806. 38095808 10.1007/s40121-023-00895-x PMC10746655

[59] Nunes A, Abreu A, Furtado B, Soares da Silva A, Coelho EB, de Barros EN. Epidemiology of pertussis among adolescents, adults, and older adults in selected countries of Latin American: a systematic review. Hum Vaccin Immunother 2021; 17:1733–46. 33734002 10.1080/21645515.2020.1827613 PMC8115456

[60] Muloiwa R, Nicol MP, Hussey GD, Zar HJ. Diagnostic limitations of clinical case definitions of pertussis in infants and children with severe lower respiratory tract infection. PLoS One 2020; 15:e0235703. 32678857 10.1371/journal.pone.0235703 PMC7367487

[61] McClymont H, Lambert SB, Barr I, Vardoulakis S, Bambrick H, Hu W. Internet-based surveillance systems and infectious diseases prediction: an updated review of the last 10 years and lessons from the COVID-19 pandemic. J Epidemiol Glob Health 2024; 14:645–57. 39141074 10.1007/s44197-024-00272-y PMC11442909

[62] Zhao IY, Ma YX, Yu MWC, et al Ethics, integrity, and retributions of digital detection surveillance systems for infectious diseases: systematic literature review. J Med Internet Res 2021; 23:e32328. 34543228 10.2196/32328 PMC8530254

[63] Riffelmann M, Wirsing von König CH, Caro V, Guiso N. Nucleic acid amplification tests for diagnosis of *Bordetella* infections. J Clin Microbiol 2005; 43:4925–9. 16207944 10.1128/JCM.43.10.4925-4929.2005 PMC1248485

[64] Crowcroft NS, Pebody RG. Recent developments in pertussis. Lancet 2006; 367:1926–36. 16765762 10.1016/S0140-6736(06)68848-X

[65] Tan T . Summary: epidemiology of pertussis. Pediatr Infect Dis J 2005; 24:S35–8. 15876921 10.1097/01.inf.0000160910.17950.59

[66] McGirr AA, Tuite AR, Fisman DN. Estimation of the underlying burden of pertussis in adolescents and adults in southern Ontario, Canada. PLoS One 2013; 8:e83850. 24376767 10.1371/journal.pone.0083850 PMC3871538

[67] Masseria C, Krishnarajah G. The estimated incidence of pertussis in people aged 50 years old in the United States, 2006–2010. BMC Infect Dis 2015; 15:534. 26584525 10.1186/s12879-015-1269-1 PMC4653927

[68] Araya S, Perez T, Troche A, et al COVID-19 y coberturas de vacunación del calendario regular del Paraguay, efecto de la pandemia. Pediatría (Asunción) 2021; 48:162–8.

[69] Mongua-Rodríguez N, Delgado-Sánchez G, Ferreira-Guerrero E, et al Vacunación en menores de cinco años. Salud Publica Mex 2024; 66:368–80. 39977087 10.21149/15793

[70] World Health Organization . Diphtheria tetanus toxoid and pertussis (DTP) vaccination coverage. 2024. Available at: https://immunizationdata.who.int/global/wiise-detail-page/diphtheria-tetanus-toxoid-and-pertussis-(dtp)-vaccination-coverage?CODE=URY&ANTIGEN=DTPCV1+DTPCV3&YEAR=. Accessed October 10, 2024.

[71] World Health Organization . Immunization dashboard. 2024. Available at: https://immunizationdata.who.int/dashboard. Accessed October 10, 2024.

[72] Perrett KP, Halperin SA, Nolan T, et al Immunogenicity, transplacental transfer of pertussis antibodies and safety following pertussis immunization during pregnancy: evidence from a randomized, placebo-controlled trial. Vaccine 2020; 38:2095–104. 31776029 10.1016/j.vaccine.2019.10.105

[73] Halperin SA, Langley JM, Ye L, et al A randomized controlled trial of the safety and immunogenicity of tetanus, diphtheria, and acellular pertussis vaccine immunization during pregnancy and subsequent infant immune response. Clin Infect Dis 2018; 67:1063–71. 30010773 10.1093/cid/ciy244

[74] Munoz FM, Bond NH, Maccato M, et al Safety and immunogenicity of tetanus diphtheria and acellular pertussis (Tdap) immunization during pregnancy in mothers and infants: a randomized clinical trial. JAMA 2014; 311:1760–9. 24794369 10.1001/jama.2014.3633 PMC4333147

[75] Romanin V, Acosta AM, Juarez MDV, et al Maternal vaccination in Argentina: tetanus, diphtheria, and acellular pertussis vaccine effectiveness during pregnancy in preventing pertussis in infants <2 months of age. Clin Infect Dis 2020; 70:380–7. 30877308 10.1093/cid/ciz217 PMC8876368

[76] Baxter R, Bartlett J, Fireman B, Lewis E, Klein NP. Effectiveness of vaccination during pregnancy to prevent infant pertussis. Pediatrics 2017; 139:e20164091. 28557752 10.1542/peds.2016-4091

[77] Winter K, Cherry JD, Harriman K. Effectiveness of prenatal tetanus, diphtheria, and acellular pertussis vaccination on pertussis severity in infants. Clin Infect Dis 2017; 64:9–14. 27624956 10.1093/cid/ciw633

[78] D’Heilly C, Switzer C, Macina D. Safety of maternal immunization against pertussis: a systematic review. Infect Dis Ther 2019; 8:543–68. 31531826 10.1007/s40121-019-00265-6 PMC6856234

[79] Nishiyama H, Tajiri T, Kurokawa R, et al Prevalence and clinical relevance of comorbid pertussis infection in adult patients with asthma: a prospective, cross-sectional study. Respir Investig 2024; 62:811–6. 10.1016/j.resinv.2024.07.006 39018657

[80] Chen J, Shin JY, Kim H, et al Incidence and healthcare burden of pertussis among older adults with and without pre-existing chronic obstructive pulmonary disease or asthma in South Korea. COPD 2023; 20:126–34. 37093711 10.1080/15412555.2023.2169120

